# Strategic planning for reform of human resources for the supply chain within Mozambique’s health system

**DOI:** 10.1186/2052-3211-7-S1-O5

**Published:** 2014-12-17

**Authors:** Kevin Pilz, Paulo Nhaducue, Deogratias Gasuguru

**Affiliations:** 1 USAID/Mozambique, Maputo, Mozambique; 2 CMAM, Ministry of Health, Maputo, Mozambique; 3 SCMS, John Snow Inc., Maputo, Mozambique

## Background

The Ministry of Health (MOH) of Mozambique became a People that Deliver (PtD) focus country in 2011. Soon after the PtD Conference in 2011, the MOH re-ignited the development of a Strategic Plan for Pharmaceutical Logistics and decided to make reform of Human Resources for the Supply Chain (HR for SC) a key pillar of the plan, as well as a component of the Health Sector Strategic Plan (2014-2019). Both Strategic Plans were approved in 2013, and the corresponding Logistics Implementation Plan was developed in 2014.

## Method

The HR for SC component of the Logistics Strategic Plan was developed utilizing existing documentation from Mozambique and PtD. The MOH, USAID and SCMS then conducted a qualitative situational assessment of the human resources for SC, as the basis for developing the Implementation Plan. The assessment was adapted from USAID | DELIVER PROJECT’s Human Resource Capacity Development Assessment Guide and Tool, and involved a participative process with staff from CMAM, the MOH’s Human Resources Directorate, provincial warehouses, and partner organizations.

## Results

The specific objective for HR in the Logistics Strategic Plan is defined as: Sufficient trained, qualified, experienced and motivated personnel are available, and conditions exist that permit their retention, at all levels of the supply chain. The main strategies outlined in both the Logistics and Health Sector Strategies are: understanding the competencies and personnel required at all levels of the supply chain, creating supply chain-specific educational degrees and cadres, strengthening and coordinating the in-service and pre-service logistics training of health cadres through National Health Institutions, and developing mechanisms for improved retention for supply chain staff within the public sector.

The HR for SC assessment resulted in 18 recommendations and over 60 suggested activities, which were transformed directly into a prioritized and budgeted Implementation Plan. The MOH and partners are currently mobilizing resources to fund these activities.

## Discussion

The MOH of Mozambique recognizes that strategic efforts to improve access to medicines must include a dedicated effort to establish and institutionalize national systems to fulfil the human resource requirements of the health supply chain at all levels. As such, Mozambique is probably one of the first countries to incorporate reforming HR for SC within their Sector-Wide Health Strategy. Implementation of the Mozambique’s new Strategic Plans, with support from partners, will be essential to achieve Mozambique’s long term goals, both in terms of supply chain performance and health outcomes.

## Lessons learned

• The attention brought to HR for SC by the People that Deliver Initiative and Mozambique’s involvement as a focus country were critical in assuring that the Government’s strategic plans focus on new approaches within this key area.

• The process of developing a costed and prioritized Implementation Plan to reform HR for SCM was greatly facilitated by first conducting a participatory assessment of the challenges and opportunities in the area.

